# Seizure-6 proteins highlight BACE1 functions in neurobiology

**DOI:** 10.18632/oncotarget.13801

**Published:** 2016-12-05

**Authors:** Martina Pigoni, Jenny M. Gunnersen, Stefan F. Lichtenthaler

**Affiliations:** German Center for Neurodegenerative Diseases, Neuroproteomics, Klinikum rechts der Isar, Technische Universität München, Munich Cluster for Systems Neurology and Institute for Advanced Study, Technische Universität München, Munich, Germany

**Keywords:** Alzheimer’, s disease, beta-secretase, biomarker, dendritic spines, excitatory synapse development

Proteins of the Seizure protein 6 (Sez6) family are abundant in neurons and are localized to the somatodendritic compartment of these highly polarised cells (Gunnersen et al., 2007; Miyazaki et al., 2006). Two recent studies (Kuhn et al., 2012; Pigoni et al., 2016) highlight the important role of the Alzheimer’s disease (AD)-linked aspartyl-protease β-site amyloid precursor protein (APP) cleaving enzyme 1 (BACE1) in the regulation of Sez6 and Sez6-like (Sez6L), influencing their sub-cellular distribution and, presumably, their function.

**Figure 1:**
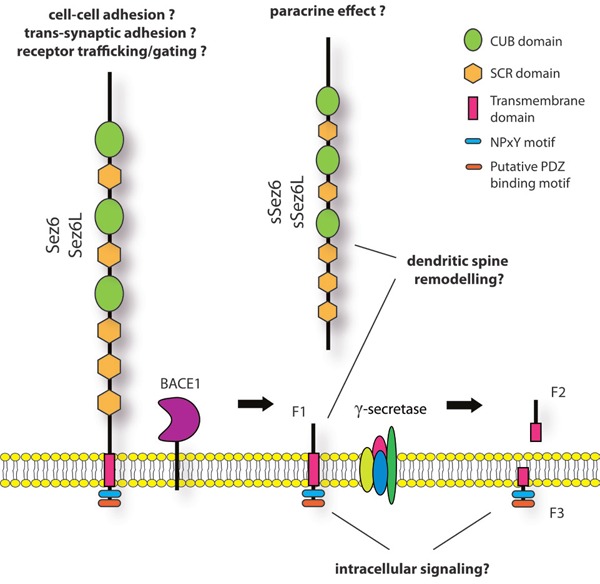
Schematic representation of Sez6 and Sez6L structure and proteolytic processing Sez6 proteins contain three CUB domains, five SCR domains and a NPxY motif. A PDZ binding domain has been predicted in the C-terminal part of the protein. Sez6 and Sez6L are cleaved by BACE1 in the brain, resulting in a secreted, soluble ectodomain (sSez6, sSez6L) and a C-terminal, membrane-bound fragment (F1). F1 is further processed by γ-secretase which is expected to lead to two smaller fragments, F2 and F3. When γ-secretase is blocked, F1 accumulates.

The three members of the Sez6 family of proteins, which also includes Sez6L2, are single transmembrane domain proteins with large extracellular regions (Figure 1). The presence of multiple CUB and SCR (also termed CCP or sushi) domains suggests adhesive and/or receptor trafficking functions of these proteins, however their binding partners are not yet known. Sez6 proteins may trigger intracellular signaling via their cytoplasmic NPxY motif, a phosphotyrosine-binding domain (PTB)- containing protein interaction motif that also mediates internalization from the cell surface. A similar motif in another BACE1 substrate, APP, controls access of APP to endosomally-localized BACE1. Also similar to APP, the Sez6 intracellular domain is released from the membrane by γ-secretase (Pigoni et al., 2016) through “regulated intramembrane proteolysis”.

All three of the Sez6 family members were initially identified as candidate substrates for BACE1 in neurons using the proteomic SPECS technique (Kuhn et al., 2012). The recent demonstration that Sez6 and Sez6L shedding is essentially abolished by BACE inhibitors as well as in BACE1 knockout and BACE1/2 double knockout mice, validates these proteins as *bona fide* BACE1 substrates in the brain (Pigoni et al., 2016).

The discovery and validation of Sez6 and Sez6L as BACE1 substrates is noteworthy for several reasons. Firstly, unlike APP, Sez6 and Sez6L are “exclusive” substrates of BACE1, meaning that other proteases do not compensate when BACE1 function is inhibited. Therefore, levels of the BACE1-shed forms of these proteins in cerebrospinal fluid (CSF) could provide a direct readout of BACE1 activity. Clinical trials to determine the efficacy of BACE inhibitors as AD therapeutics are currently underway, based on evidence that blocking BACE1 activity reduces the production of the Aβ peptide from APP and the associated deleterious effects on neurons. While no serious adverse effects of BACE inhibition have been reported so far, the shedding of Sez6, Sez6L and the numerous additional substrates of BACE1 will also be blocked and, potentially, cause side-effects. Thus, measuring CSF Sez6 and Sez6L levels in addition to Aβ may allow i) a precision medicine approach, where patients are dosed individually in order to maximize Aβ reduction while minimizing effects on other BACE1 substrates, and ii) development of a new generation of substrate-specific BACE1 inhibitors. These considerations are particularly relevant given that inhibitors for the other Aβ-generating protease, γ-secretase, failed in previous clinical trials because they also blocked signalling of another γ-secretase substrate, Notch.

Secondly, while BACE1 seems to be enriched in axons, with immunostaining being particularly strong in the mossy fibres of dentate gyrus granule cells in the hippocampus (Kandalepas et al., 2013)-, Sez6 and Sez6L are somatodendritic and exhibit more generalized expression throughout the brain than immunoreactive BACE1. Thus, the identification of these two “exclusive” BACE1 substrates will stimulate increased efforts to resolve the so-called “spatial paradox” and to co-localize BACE1 and its substrates.

Third, BACE1 activity fine-tunes the balance between cell-surface and shed forms of Sez6 proteins and may, thus, be implicated in regulating structural and functional neuronal plasticity. While there is still much to be learned about Sez6 protein signalling mechanisms, gene knockout studies in mice have highlighted their important roles in excitatory synapse development and function (Gunnersen et al., 2007) and synaptic circuit refinement (Miyazaki et al., 2006). Interestingly, mice deficient in either Sez6 or BACE1 show reduced dendritic spine density (Gunnersen et al., 2007; Savonenko et al., 2008), suggesting that the BACE1-shed ectodomains of Sez6 or Sez6L participate in retrograde and/or paracrine signalling and promote synapse formation and function. Alternatively, the membrane-bound fragment remaining after BACE1 cleavage may be relevant, similar to recent findings on CHL1, another BACE1 substrate (Barao et al., 2015). The observation that BACE1 serves to down-regulate surface levels of Sez6 family proteins (as surface levels increase when BACE1 is blocked; (Pigoni et al., 2016) could indicate an activity-dependent proteolytic control of dendritic spine morphology, similar to that seen with metalloprotease cleavage of the post-synaptic adhesion molecule Neuroligin1. Interestingly, a *sez6* gene variant has been linked to epileptic seizures. While seizures also occur in BACE1−/− mice (Savonenko et al., 2008), future research is needed to show whether this phenotype involves reduced cleavage of Sez6 or Sez6L.

Besides these roles in synapse formation and maintenance, the Sez6 family members have functions outside the nervous system. For example, polymorphisms and deletions in the *sez6L* gene and elevated Sez6L and Sez6L2 protein levels are associated with lung cancer. Whether the latter findings result from reduced proteolytic cleavage remains unclear, but they are unlikely to involve BACE1, which is expressed at lower levels in most other tissues than in the nervous system. In fact, in pancreas, all three Sez6 family members are predominantly cleaved by the BACE1-homolog BACE2 (Stutzer et al., 2013; Pigoni et al., 2016).

Taken together, the discovery and validation of Sez6 and Sez6L as “exclusive” substrates of BACE1 provides strong impetus to uncover the fundamental roles of BACE1 and its Sez6 substrates in the brain and to test the suitability of Sez6 proteins as companion diagnostics for BACE inhibitors.
